# Stigma and lack of access to quality healthcare in the transgender population

**DOI:** 10.1097/01.JAA.0000000000000083

**Published:** 2024-12-21

**Authors:** Kara Kelton

**Affiliations:** **Kara Kelton** practices at Sentara Medical Group's Center for Plastic Surgery Princess Anne in Virginia Beach, Va., and the Center for Plastic Surgery Fort Norfolk in Norfolk, Va. The author has disclosed no potential conflicts of interest, financial or otherwise.

**Keywords:** transgender, healthcare, inequality, access, stigma, LGBTQ+

## Abstract

More than 1.6 million US adults identify as transgender (that is, a gender different than the one traditionally associated with the biologic sex assigned to them at birth). These patients suffer from healthcare inequity and lack of access to healthcare, causing a public health crisis. This article seeks to raise awareness of this issue and encourage clinicians and healthcare systems to make meaningful changes to reduce healthcare stigma for transgender patients.

A transgender patient's gender identity or expression differs from the one typically associated with the biologic sex assigned to that person at birth.^1^ A cisgender patient identifies with the gender traditionally associated with the sex assigned at birth. Nonbinary patients can identify as both genders or as neither male nor female.^2^ A population study by the UCLA School of Law in 2022 found that 0.5% of all adults in the United States, or more than 1.6 million people, identified as transgender.^3^ This number is likely higher because patients do not always openly identify as transgender, and the US Census does not record gender identity. Also, research often assumes that all participants are cisgender, and transgender patients, when included, are combined with lesbian, gay, bisexual, transgender, queer, questioning, and other (LGBTQ+) identities. Lesbian, gay, bisexual, queer, and questioning are sexual minorities, and these terms describe sexual orientation, which differs from gender identity.^1^,^4^

Transgender patients suffer from marginalization, discrimination, and stigmatization in healthcare, leading to reduced healthcare access and adverse health outcomes.^1^,^5^ High-quality healthcare is known to prevent disease and improve quality of life.^6^ The healthcare inequities experienced by transgender patients cause adverse outcomes, such as a 24% decrease in preventive and emergency care.^1^,^5^ Because of these inequities, transgender patients experience an increased rate of mental health disorders such as depression, anxiety, self-harm, and substance use disorders or misuse.^1^,^7^ The transgender suicide rate is nine times that of the general population.^8^ Kachen and Pharr reviewed the 2015 US Trans Survey, showing that 30% of transgender patients (or nearly half a million people) reported healthcare discrimination.^9^ Pinnamaneni and colleagues found that transgender patients were 5% more likely not to have a primary healthcare provider, and 12% more likely to not have had a routine checkup in 5 years.^10^ Transgender patients are 39% more likely to have poor physical or mental health, which increased to 54% after the COVID-19 pandemic.^10^ The study also found that transgender patients were 8% more likely to have hypertension, 14% more likely to have cardiovascular disease, 33% more likely to have a myocardial infarction, and 24% more likely to have a cerebrovascular accident.^10^ Limitations of the data include recall bias, self-reporting of conditions, and underreporting of transgender identification in the survey.^10^

This article focuses on the healthcare inequalities experienced by transgender patients, discusses types of stigma as a root cause, and reviews the negative effects stigma has on health. Bringing these issues to light may encourage clinicians, staff, and healthcare systems to examine their practices, be more mindful, and support drastic changes in healthcare delivery.

## METHODS

A search was performed via PubMed. Resources published within the past 5 years on transgender adults in the United States were included. Sources primarily focused on HIV/AIDS, sex workers, intimate partner violence, COVID-19, gender affirmation, or mental health were excluded. This was done to prioritize the research goal of access to basic and common medical care and avoid reiterating specialized data that have been published extensively. Any article that did not differentiate between LGBTQ+ and transgender also was excluded. *Access* was used as a MeSH term and narrowed through *access to healthcare* and then *health equity*, adding *transgender* to the search criteria. This search provided 33 articles. Of the 33, none were randomized clinical trials, clinical trials, or meta-analyses. The search was repeated with MESH terms *access to healthcare* and *transgender*, which yielded 53 quality articles. Any studies considered low-quality (editorials, opinion pieces without data, and observational studies) were removed. A review of this literature produced the recurring theme of stigma in healthcare.

**Box 1:**
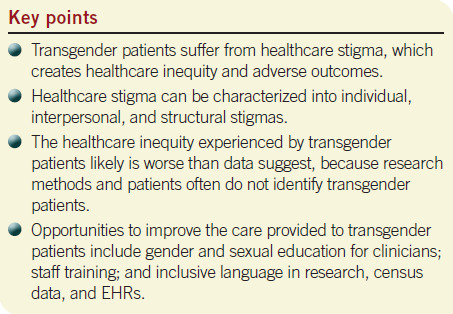
No caption available.

## DISCUSSION

Stigma, in various forms, creates health inequity in transgender patients. The Mayo Clinic defines stigma as “when someone views you in a negative way because you have a distinguishing characteristic or personal trait that's thought to be, or actually is, a disadvantage.”^11^

Velasco analyzed transgender and gender-diverse stigma in a qualitative meta-synthesis and found that stigma limited healthcare access for transgender patients and created health disparities.^12^ He also determined that understanding stigma can improve healthcare. He identified three specific levels of healthcare stigma experienced by transgender and gender-diverse patients: the individual, the interpersonal, and the structural stigma.^13^ This article reviews the manifestations of each type of stigma and the effect on the transgender community based on existing research. However, the significant lack of data and high-quality studies limits the quantitative results. The perceptive aspect of stigma and gender identity makes a double-blind, randomized controlled trial difficult, and most census data, surveys, and research categorize patients as males or females, limiting retrospective information.

### Individual stigma

This internal process is created from external experiences with a damaging effect on the health of transgender patients.^13^ Individual stigma is based on the patient's perception and represents feelings of shame, rejection, and expected adverse interactions with others.^13^ Self-worth and socialization are reduced, leading to isolation and marginalization, which prevents people from seeking healthcare.^13^ Kachen and Pharr found that 22% of people responding to the 2015 US Trans Survey delayed care because of fear of discrimination.^9^ This fear leads to minority stress, which any marginalized group can experience because of personal or cultural attitudes and reactions.^5^,^14^ This stress contributes to the increased risk of suicide and mental health issues in the transgender population. Paine noted that health is affected by these stressful experiences, particularly the acute instance of stigma and discrimination and the chronic consequences of unaddressed mental and physical health issues.^15^ A patient's individual stigma is directed internally but is shaped by experiences created by interpersonal stigma.

### Interpersonal stigma

This type of stigma is based on negative interactions, including verbal and physical abuse, with other people.^13^ Transgender patients experience more disrespect and unsafe situations in healthcare settings compared with cisgender and sexual minority patients. They may experience verbal and physical abuse and may be denied care.^1^,^13^,^15^ These experiences reduce the access to and quality of healthcare and reinforce individual stigma, which perpetuates the cycle of inadequate care.

Verbal abuse includes demeaning language, misgendering patients by using their birth gender or name, stereotyping, and inappropriate questions.^13^ Misgendering in medicine causes “embodied disruption,” when a patient's nonconformity to medical standards changes medical interactions.^15^ For example, when a patient requests preferred pronouns, a negative response from a clinician results in the reduction or cessation of medical care.^15^ Transgender patients are twice as likely to suffer from verbal abuse from healthcare providers compared with sexual minority patients.^1^ Paine performed a qualitative study on the healthcare experiences of 34 adult LGBTQ+ women, transgender men, and nonbinary patients.^15^ The study conducted narrative interviews; embodied disruption was a prominent theme in all groups.^15^ Kcomt also demonstrated healthcare inequities following stigma.^1^ A systematic review, including eight studies of 43,570 transgender and LGBTQ+ patients, found that transgender patients are more medically compromised than sexual minority patients.^1^

Physical abuse also is more common in transgender patients, including unnecessary medical procedures or medication, withholding care, and privacy violations.^1^,^16^ Per Kcomt, transgender patients are twice as likely to be physically abused by a healthcare provider, including being physically attacked in a healthcare setting, compared with sexual minority patients.^1^ Kcomt also found that transgender patients were more than three times as likely as their sexual minority counterparts to be denied care.^1^ Vupputuri and colleagues analyzed focus group sessions with transgender patients, and of the six themes discovered, interpersonal stigma covered half.^16^ These included negative experiences with clinicians, issues with case management, and negative experiences with staff.^16^ Interpersonal stigma stems from interactions between persons, and structural stigma forms when that interaction is seen on a systemic level.

### Structural stigma

Individual ignorance creates interpersonal stigma between patients and clinicians; however, widespread clinician and staff ignorance creates a systemic or structural stigma. This stigma is created and maintained by cultural gender norms and the perpetuation of barriers to care, reinforcing the individual stigma.^13^ Structural stigma is born from an asymmetric power relationship between the patient and the healthcare provider. Patient mistrust in clinicians and staff causes delays or complete lack of medical treatment.^5^ The healthcare cultural incompetence causes patients to seek out unlicensed healthcare providers for illicit medical care or procedures.^1^,^13^ Structural stigma leads to adverse healthcare outcomes by reducing care quality, thoroughness, appropriateness, and timeliness. It can worsen acute and chronic illnesses and lead to new health issues.^5^

Clinicians often are unprepared to care for transgender patients because of a lack of medical and cultural training, creating a healthcare barrier.^1^,^9^,^17^ Few medical programs offer a transgender or LGBTQ+ curriculum, leaving clinicians ill-equipped to care for this population.^1^,^9^,^17^ A study by Harvard Medical School found that medical students in the United States receive an average of 5 hours of sexual and gender minority education.^17^ In 2018, the medical school started a 3-year sexual and gender minority health equity initiative, which is ongoing.^17^ Ufuah and colleagues also discussed the need for cultural competency programs in medical schools as part of a multidisciplinary plan to improve medical care.^18^ Studies have shown that discrimination, fear, and mistrust leads to reduced cancer screening and lack of knowledge by oncologists about high-risk cancers in transgender and sexual minority groups.^18^ Shires and colleagues found that only 85.7% of the 308 primary care providers interviewed in Midwest Health System would be willing to care for a transgender patient and even less, 78.6%, would perform a Papanicolaou test.^19^ Vupputuri and colleagues compared 282 transgender and 2,370 cisgender patients using the Kaiser Permanente transgender registry.^16^ They found increased virtual visits, cancelations, no-shows, and behavioral health visits in the transgender group, with a reported lack of clinician knowledge about transgender patients as the most significant contributor.^16^ The training deficit also is seen in healthcare support staff. Redcay and colleagues found that clinical staff lacked training, creating distrust and adverse health outcomes.^20^ Sileo and colleagues performed a qualitative study and found that transphobia, knowledge deficits, gossip, and misgendering caused healthcare workplace stigma.^21^

Healthcare systems also suffer from stigma. Nair and colleagues found three main themes when they analyzed LGBTQ+ and transgender patient experience issues.^22^ The lack of clinician knowledge and patient-centered care has been established; however, they also found a lack of institutional infrastructure to provide affirming care.^22^ Many electronic health record (EHR) systems do not include transgender patients; they use polarizing gender terms, which Ram and colleagues call “EHR-mediated violence.”^23^ EHRs have complex and erroneous diagnostics and billing concerning transgender patients.^23^ Redcay and colleagues found a lack of standardized intake procedures that often do not gather gender identity.^20^ Sileo and colleagues also noted that healthcare systems do not have enforceable policies to reduce stigma.^21^ Ng and colleagues changed the intake process at a clinic in 2020 to be more inclusive, sensitive, and respectful.^4^ The clinic hired an LGBTQ+ clinical coordinator to direct new patients, added pronoun preference to intake forms, and offered a telehealth option for new patient visits. After the changes, all 166 patients surveyed reported positive experiences.^4^

Research is another system that poorly represents transgender patients; it is narrowly focused on HIV as related to transgender and LGBTQ+ patients.^4^ Recently, research into LGBTQ+ and transgender patients has increased; however, most studies remain focused on HIV, rather than the disproportionate increases in suicide and hypertension among transgender patients.^20^ Lund and Burgess stated that the lack of data on transgender patients complicated evidence-based medical treatment of the population.^5^ The medical system as it stands is incapable of appropriately evaluating data from medical records because the gender options are typically only male or female, and therefore researchers unable to extrapolate transgender population data.

## CONCLUSION

The transgender population is suffering from health inequity because stigma causes a lack of access to quality healthcare. Stigma creates disparities and can be seen in individual, interpersonal, and structural contexts.

The limited data on transgender healthcare show increased disease and mental health disorder rates, with reduced cancer screenings and clinician knowledge. The incidence of suicide, coronary artery disease, and cancer, among other disorders, need to be examined in transgender patients to improve our understanding of stigma in healthcare. In a world of evidence-based medicine, transgender data are sparse, and without studies using multiple gender identities, data will continue to be limited. The World Health Organization also recognizes health inequity in the transgender population and in December 2023 published healthcare guidelines to increase “access and utilization of quality and respectful health services” for the transgender population.^24^ Considering that more than 1 million US patients identify as transgender, the healthcare inequity experienced by this population could be viewed as a public health crisis.

Personal and institutional changes are needed to provide better care for the transgender population. Clinicians must be made aware of health concerns and challenges that face transgender patients, the best practices, and be mindful of additional environmental factors that affect health outcomes.^4^,^13^,^15^,^16^ All medical schools should incorporate transgender and sexual minority groups into the curriculum. Students are taught to discuss code status and give terminal diagnoses and death notices; learning how to speak to a transgender patient also should be included in training.

As a system, access to quality healthcare requires that healthcare organizations implement inclusive and respectful procedures. Healthcare teams must be culturally competent, appropriate communicators, and understand patients' individuality to provide quality care.^18^ This includes more inclusive language in person and EHR systems by using correct pronouns and names, updating gender identity, and making gender identity a common intake question. Medical staff also must be trained in appropriate language, with systems instituting policies that penalize those perpetuating the stigma. Small continuous changes may be easier to implement and change culture over time, such as yearly modules and competencies to improve care.

These changes are challenging to implement because the information is limited, and more research is needed to identify the unique needs of transgender patients.^22^ This article is limited by the lack of studies, surveys, and published transgender-inclusive practices. Adding a gender identity question to the US Census or conducting nongender-specific research will add significant retrospective data. Research aimed at the health issues faced by transgender patients is needed to reduce inequity and fuel evidence-based practice. Additional studies are required to determine the most effectual modifications and if those modifications make a significant difference in addressing these concerns. The 2022 US Trans Survey is reviewing collected data and will have additional up-to-date data soon, but more research and communications are needed.^25^

The medical community must continue to make drastic changes to provide high-quality, respectful, and inclusive care. Patients, clinicians, and staff must be mindful and supportive of transgender patients. Reducing interpersonal stigma, one person at a time, with appropriate interactions, will reduce each patient's individual stigma. To make institutional changes, the medical community must agree that the current treatment of the transgender population is contrary to our healthcare mission, and we must rectify our practices to provide appropriate care.
